# Deep dry needling of trigger points located in the lateral pterygoid muscle: Efficacy and safety of treatment for management of myofascial pain and temporomandibular dysfunction

**DOI:** 10.4317/medoral.20384

**Published:** 2015-02-07

**Authors:** Luis-Miguel Gonzalez-Perez, Pedro Infante-Cossio, Mercedes Granados-Nunez, Francisco-Javier Urresti-Lopez, Ricardo Lopez-Martos, Pablo Ruiz-Canela-Mendez

**Affiliations:** 1 Department of Oral and Maxillofacial Surgery Virgen del Rocio University Hospital, Seville, Spain

## Abstract

**Background:**

To determine whether deep dry needling (DDN) of trigger points (TPs) in the lateral pterygoid muscle (LPM) would significantly reduce pain and improve function, compared with methocarbamol/paracetamol medication.

**Material and Methods:**

Forty-eight patients with chronic myofascial pain located in the LPM were selected and randomly assigned to one of two groups (DDN test group, n=24; drug-treated control group, n=24). The test group received three applications of needling of the LPM once per week for three weeks, while control group patients were given two tablets of a methocarbamol/paracetamol combination every six hours for three weeks. Assessments were carried out pretreatment, 2 and 8 weeks after finishing the treatment.

**Results:**

A statistically significant difference (*p*<0.05) was detected for both groups with respect to pain reduction at rest and with mastication, but the DDN test group had significantly better levels of pain reduction. Moreover, statistically significant differences (*p*<0.05) up to day 70 in the test group were seen with respect to maximum mouth opening, laterality and protrusion movements compared with pretreatment values. Pain reduction in the test group was greater as a function of pain intensity at baseline. The evaluation of efficacy as assessed both by patients/investigators was better for the test group. 41% of the patients receiving the combination drug treatment described unpleasant side effects (mostly drowsiness).

**Conclusions:**

DDN of TPs in the LPM showed better efficacy in reducing pain and improving maximum mouth opening, laterality, and protrusion movements compared with methocarbamol/paracetamol treatment. No adverse events were observed with respect to DDN.

**Key words:** Myofascial pain syndrome, myofascial trigger points, deep dry needling, lateral pterygoid muscle, randomized controlled trial, temporomandibular disorders.

## Introduction

Myofascial pain syndrome (MPS) is a complex disorder of the musculoskeletal system with multi factorial involvement and diverse clinical presentations in several areas of the body, one of them being in the orofacial region with involvement of the temporo-mandibular joint (TMJ) and masticatory muscles (1). MPS must be suspected in patients with pain in the masticatory muscles, along with the existence of painful trigger points (TPs) on palpation, and limitation of interincisal opening (2). One of the masticatory muscles most frequently affected is the lateral pterygoid muscle (LPM) (3), and deep dry needling (DDN) is one of the techniques used for treating. Its purpose is to inactivate the TPs (4), this being an essential outcome for the effective management of this pathology. Literature reports attest to the safety, efficacy and low cost of this treatment approach (5-12).

The objective of the present study was to investigate whether DDN of the LPM could reduce pain and enhance mandibular mobility compared with administration of a methocarbamol/paracetamol combination treatment. Secondary objectives were to assess the level of improvement in the general state of the TMJ, as well as to assess patient tolerance to the treatments followed, and side effects reported.

## Material and Methods

- Subjects

An open, randomized, single center clinical trial was carried out at the Outpatient Clinic of the Department of Oral and Maxillofacial Surgery at the Virgen del Rocio University Hospital, Seville (Spain), between May and October 2013. Male and female patients between 18 and 65 years of age with temporo-mandibular myofascial pain located in the LPM were enrolled following confirmation of MPS according to clinical signs of Simons (4,13) and imaging results, panoramic radiography and magnetic resonance imaging (MRI), to rule out the presence of other conditions. We included patients with temporo-mandibular myofascial pain of more than six months’ duration only or with moderate limitation of mandibular movement (interincisal opening limited to <40 mm and passive stretching required to force the opening by ≥ 5 mm, according to Group I criteria of the International RDC-TMD Consortium (14), with the presence of TPs in the LPM. The following inclusion diagnostic criteria were assessed: a) strong pain in the anterior part of the lower belly of the LPM on palpation; b) deep-seated pain in the TMJ and/or region of the maxillary sinus (referred pain); and c) significant motor dysfunction (limited jaw opening, painful protrusion of the chin against resistance, mandibular lateralization to the opposite side upon opening). Subjects were excluded if they presented with one or more of the following conditions: TMJ internal derangements with anterior disk displacement without reduction, degenerative joint disease, history of jaw trauma, vascular diseases, migraine and tension headaches, and history of infectious-inflammatory conditions of odontogenic origin. If another TP can be detected in any of the other elevator muscles, these areas were inactivated before DDN of TP in the LPM, so that 8 patients of our study with TPs also in masseter muscle received, in addition to treatment with DDN, an adjuvant treatment with deep tissue massage and manual stretching of the masseter muscle in order to prepare the treatment area (LPM) by muscle relaxation of adjacent structures.

The Committee for Research and Clinical Ethics of the Hospital approved the study (2013PI/119). Declaration of Helsinki guidelines was followed. Before inclusion, all patients signed an informed consent form.

- Study Design

**Figure 1:**
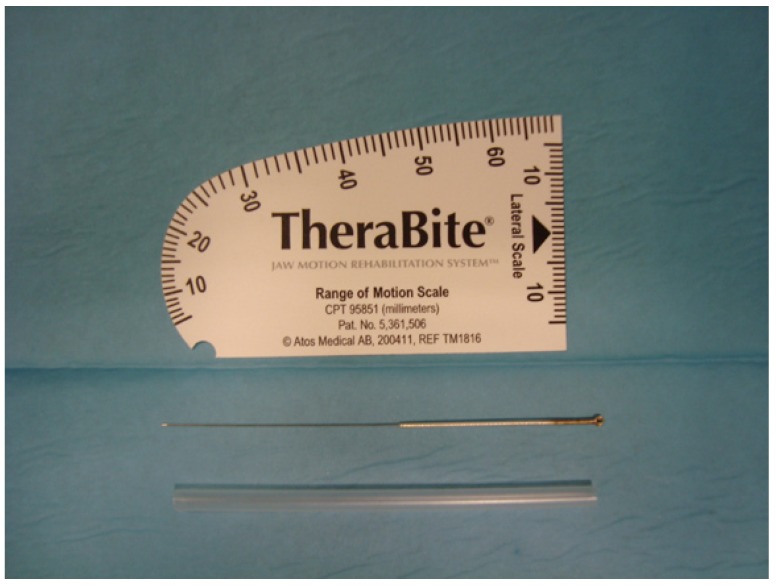
Range of mandibular movements was measured with a Therabite® ruler. For the DDN, sterile stainless steel needles (length 40 mm and caliber 0.25 mm, with a cylindrical plastic guide; Agu-punt ®) were used.

**Figure 2:**
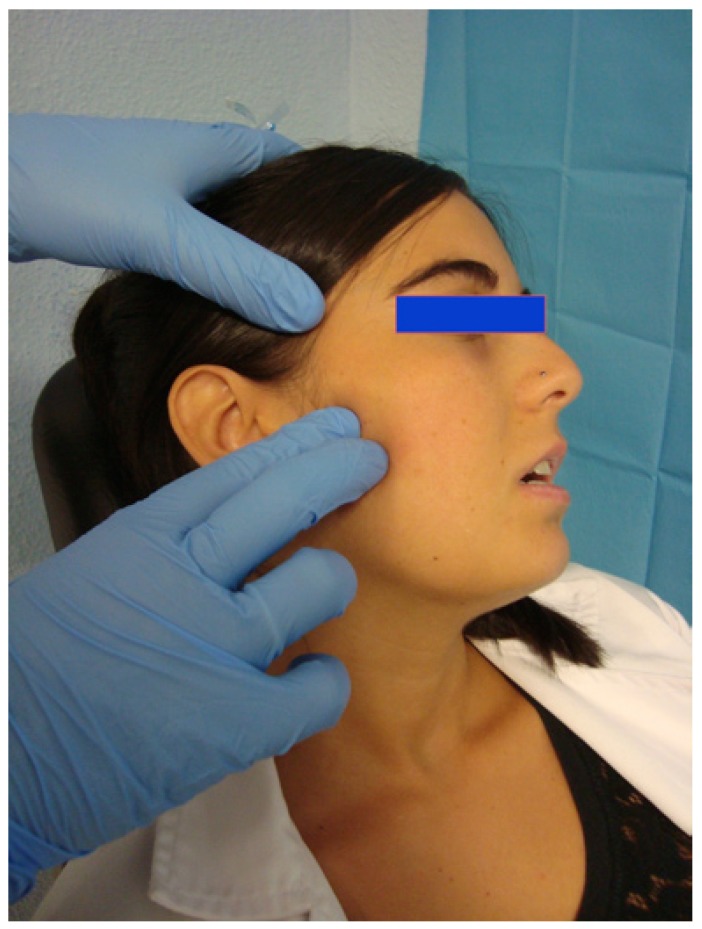
Anatomic location of the lateral pterygoid muscle for needling.

**Figure 3:**
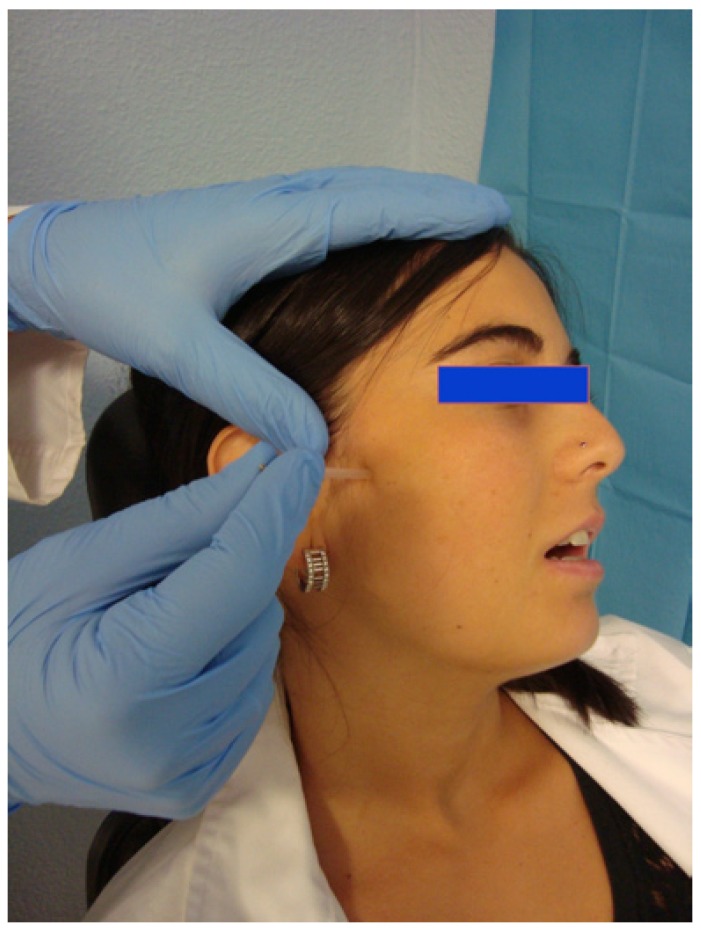
The needle was inserted taking into account the relationship between the muscle and TPs with the surrounding anatomical structures. The presence of a local twitch response during DDN is important given its proven relationship with the desired therapeutic effect.

Patients were assigned randomly to one of two groups (Epidat 4.0). The DDN group received needling of the LPM once per week for 3 weeks. Clinical evaluations were carried out at the basal day (day 0), and on days 28 and 70 after the commencement of treatment. Data were collected at each visit by a same observer. For the DDN therapy, sterile stainless steel needles (length 40 mm/ caliber 0.25 mm, with a cylindrical plastic guide; Agu-punt ®) were used (Fig. 1). The preauricular area was swabbed with 90° alcohol, and the LPM manually located intra and extra-orally, unilaterally (Fig. 2). Intramuscular needling was then carried out. This was performed via a deep puncture into myofascial pain TPs without the introduction of any substance (dry needling) (Fig. 3) (4,15). The aim was to provoke a jump reaction or local twitch response (LTR) in all cases when the needle was inserted into a TP (3,15,16). The procedure was followed by compressive haemostasis for one minute.

The control group was given a methocarbamol (380 mg) and paracetamol (300 mg) combination drug therapy, at a dose of two tablets every six hours for three weeks. For the control group, clinical evaluations were conducted at the basal day (day 0), and on days 28 and 70 following commencement of the study. Data were collected at each visit by a same observer.

- Measures

The main parameters to assess the effectiveness of the treatment were: 1) pain at rest and upon mastication using the visual analogue scale (VAS 10 cm), and 2) range of mandibular movements associated with opening of the mouth, lateral movements and protrusion measured with a Therabite® ruler. Signs that were evaluated as indicators of the effectiveness of the DDN were: significant reduction in myofascial pain at rest and with mastication, recovery of normal ranges of mouth opening, lateral and protrusive movements, and improved performance of the TMJ. In addition, TMJ affectation was assessed using a questionnaire consisting of a 100-point scale (0 worst state, 100 optimum state) based on pain (maximum 40 points), function (45 points) and mastication (15 points). Secondary efficacy outcomes were overall efficiency ratings assessed by the patient and the authors using a 5-point scale ranging from worst-0 to optimum-4. Tolerability to the treatment was evaluated by the patient and the authors using a 5-point scale (0-very bad, 1-bad, 2-acceptable, 3-good, 4-excellent). The type and frequency of adverse events were recorded at each visit.

- Statistical Analysis

Data were analyzed with statistical software (IBM SPSS Statistics 19.0). Longitudinal data were analyzed first with an overall statistical test (Friedman test). Absolute and relative frequencies were used in the case of qualitative variables. The Shapiro-Wilk test (n<50) for normality was used for quantitative variables, which were expressed as average and standard deviation (SD) or as the fiftieth percentile (P50; median), or P25-P75 (interquartile range). Non-normally distributed data were analyzed with non-parametric tests. Pre- and post-intervention comparisons of the variables in each group were performed using the Friedman test and the Wilcoxon signed ranks test (with Bonferroni correction) to analyze intra-group variations between day 0 and day 28 and between day 0 and day 70. Comparisons between the study groups were carried out with the Mann-Whitney U-test for each time point. Values of *p*<0.05 were considered to indicate statistical significance.

## Results

Forty-eight patients were selected and randomized to the study (DDN test group, n=24; drug-treated control group, n=24). Both groups had identical male-to-female ratios (5 males: 19 females), and similar age distributions (mean 34.3 SD ± 13.8 years in the DDN group vs. 35.5 ± 11.2 in the control group). In the DDN group, the right and left TMJ were affected in 12 cases each; in the control group, the right TMJ was affected in 16 of the 24 patients and the left TMJ in the remainder. Radiographic findings showed no alteration in the morphology of the articular surfaces. MRI studies revealed no significant changes. All patients from DDN group completed the trial, whereas in the drug group 8 patients terminated the study prematurely in the review phase (not receiving medication at that time).

Data from the tables are the median of the differences between the different days. From day 0 to day 70, the median pain score at rest in the DDN group decreased a rate of 68%, and 63% for the control group. For each group, the reduction in pain at rest was statistically significant on days 28 and 70 with respect to day 0, while the reduction of pain at rest produced in both groups was significantly better in the DDN group compared with the control group on day 28 (*p*=0.005) and on day 70 (*p*=0.016) ( Table 1).

**Table 1:**
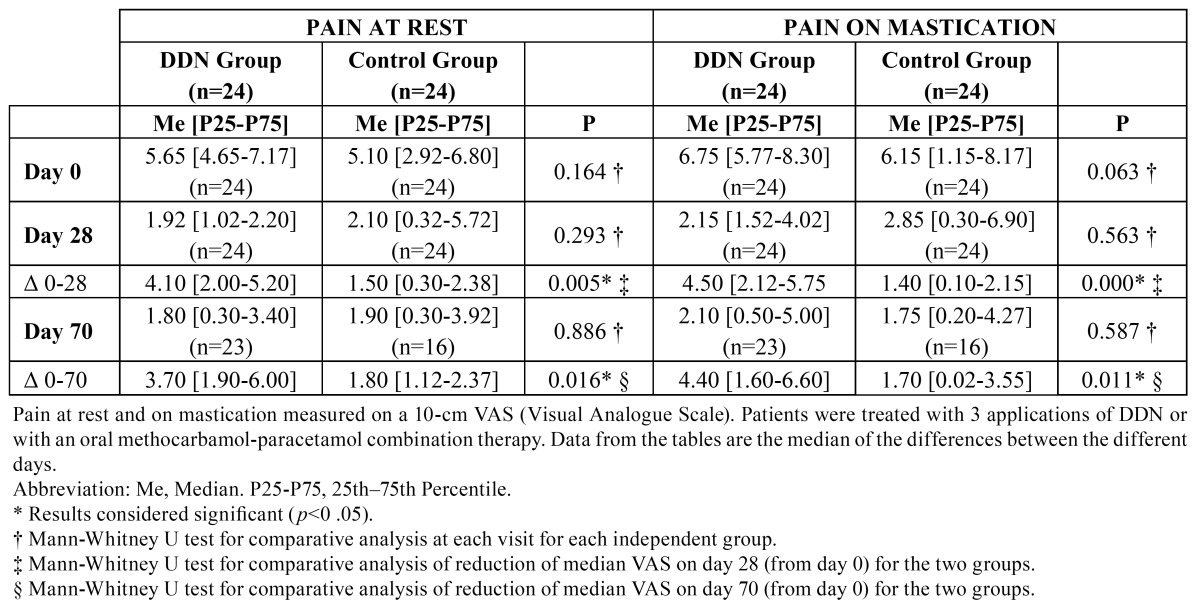
Pain at rest and on mastication.

From the beginning of the study to day 70, mastication-associated pain decreased, in DDN and control groups, a rate of 69%, and 72% respectively. The pain reduction with mastication was statistically significant at day 28 for both groups, and on day 70 for the DDN group alone. A comparison of the reduction in pain produced in both groups highlighted a statistically significant greater reduction of pain in the DDN group compared with the control group on day 28 (*p*<0.001) and day 70 (*p*=0.011) ( Table 1).

The DDN group showed improvements in the maximum mouth opening, left side, right side and protrusion movements from day 0 to day 70; these increased, respectively, a rate of 2%, 38%, 29% and 40%. Patients in the control group showed improved median movements involving opening of the mouth and displacement to the left side on day 70 with respect to day 0, increasing, respectively, a rate of 13.75% and 13%. However, in the control group, the range of right side lateral movement showed no variation on day 70 with respect to day 0, and the protrusion distance decreased 20% on day 70. Improvement in the movements of maximal mouth opening, left side, right side lateral movements and protrusion was statistically significant only for the DDN group on days 28 and 70. Mandibular protrusion in the DDN group data was significantly better than in the control group on day 28 (*p*=0.031) and day 70 (*p*=0,001). A comparison of the change in each of the mandibular movements seen in each group on days 28 and 70 showed that the movements of left side, right side and protrusion in the DDN group were statistically significantly improved compared with the control group, but no significant improvement in maximum opening was seen ( Table 2, Table 3, Table 4).

**Table 2:**
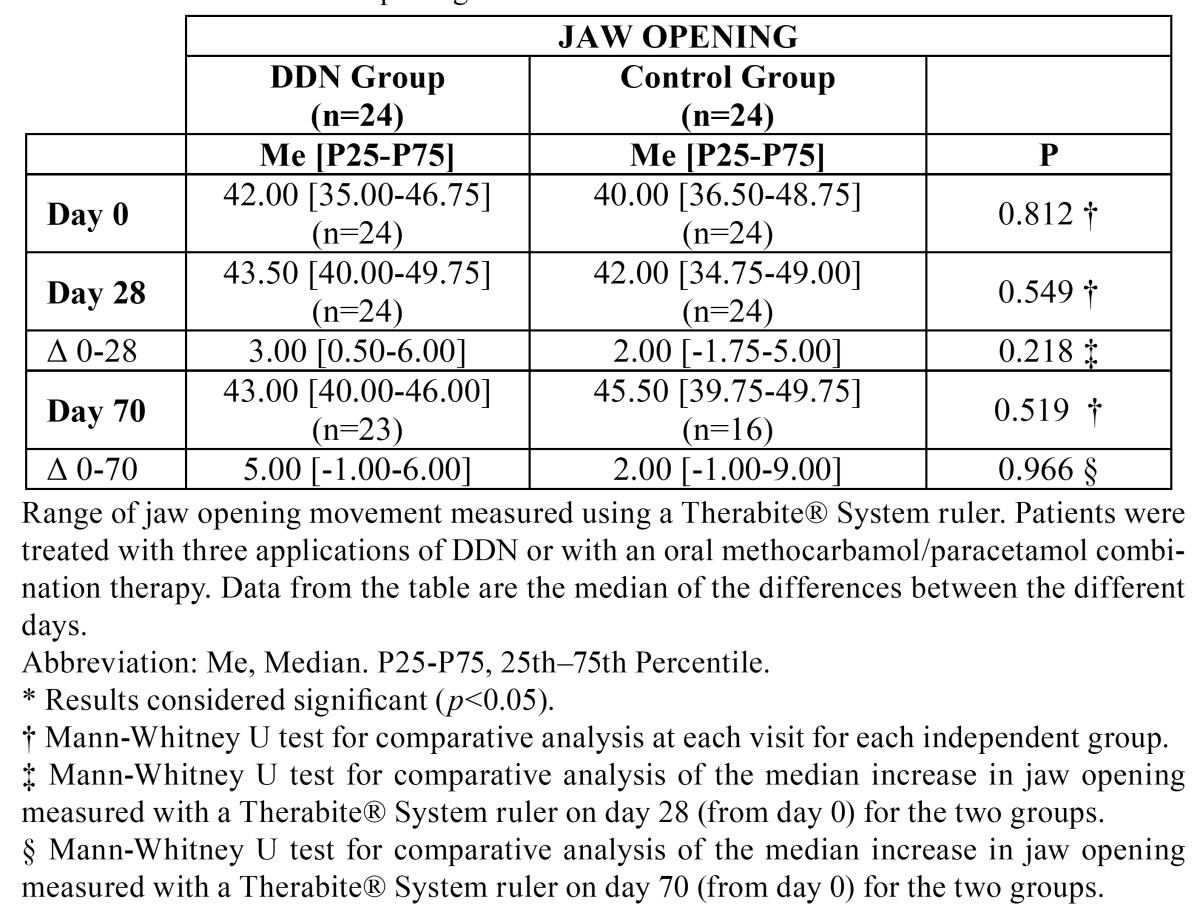
Jaw opening.

**Table 3:**
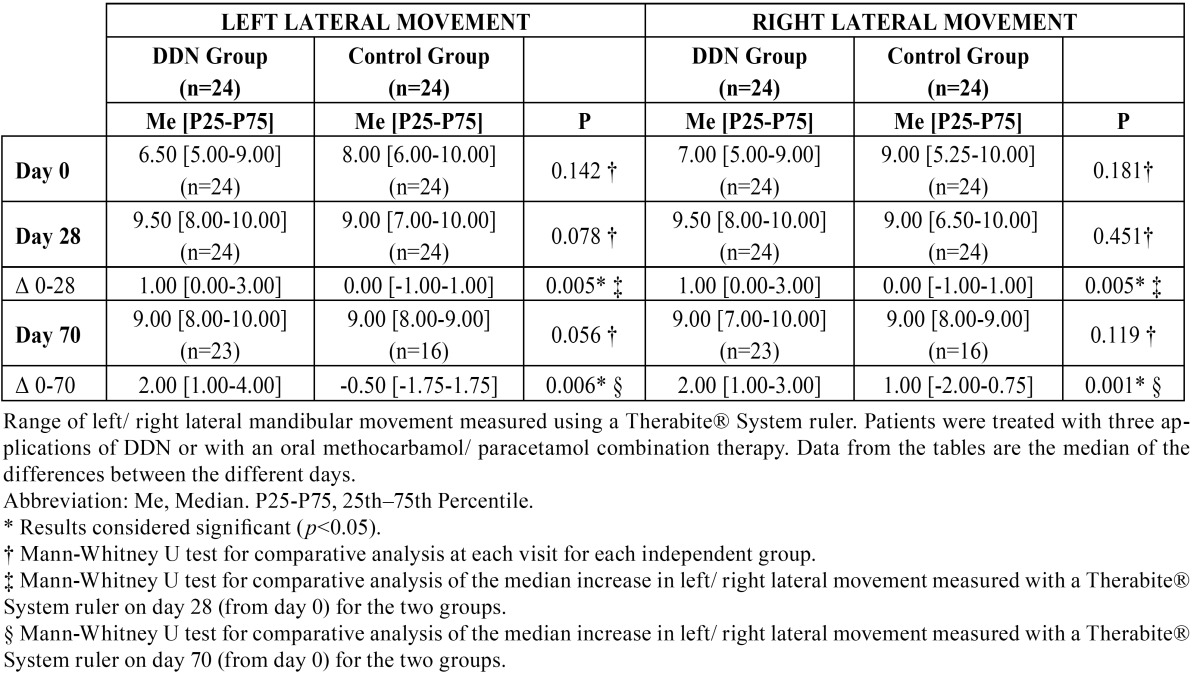
Left and right lateral movements.

**Table 4:**
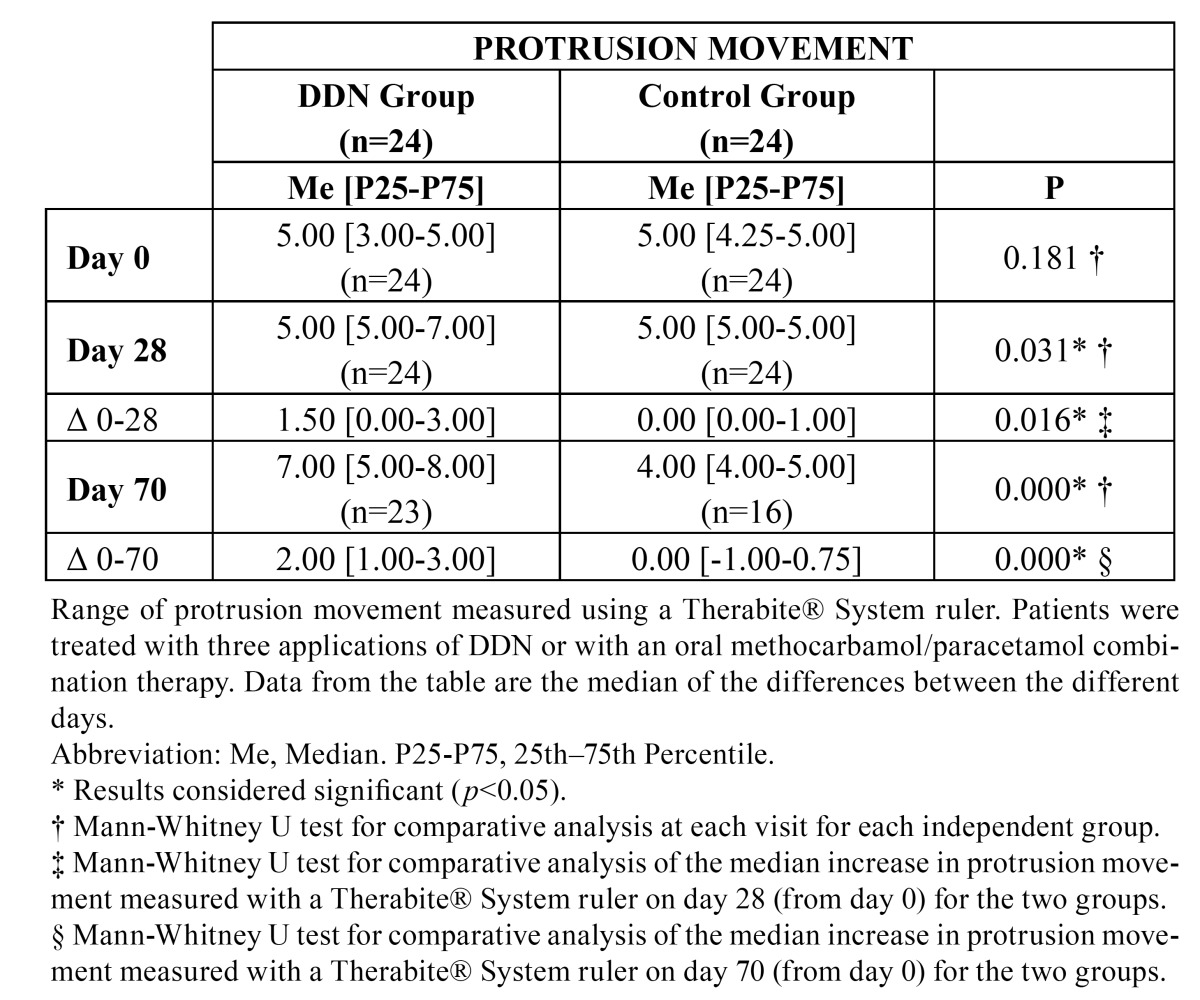
Protrusion movement.

An evaluation of the functionality of the TMJ showed that median scores in the DDN group improved almost a rate of 56%, from the beginning of the study to day 70. In the control group, there was an improvement in the median score with an increase of 35%. The improvement in the TMJ functionality was statistically significant on days 28 and 70 of the study for both groups. The change in scores that took place from days 0-28 and 0-70 of the study showed a statistically significant improvement for the DDN group compared with the control group (*p*=0.027) with regards to the day 28 score, but not the day 70 score. We also observed a statistically significant difference (*p*=0.030) between the initial scores for both groups (day 0), but this was not relevant from a clinical point of view because patients were randomly assigned to groups.

Patients in the DDN and control groups considered the outcome of their treatment to be optimal in 42% and 13% of cases, respectively, while for the authors these values were 50% and 8% for the respective groups. Tolerance to the treatment was evaluated by patients as excellent in 25% and 4% for the DDN and control groups, respectively. No patients in the DDN group exhibited side effects on any of the days of the study, while 10 patients (41%) in the control group reported side effects related to drug therapy (consisting mainly of drowsiness).

## Discussion

Clinical guidelines related to the treatment of TMD recommend the management of myofascial pain from a multidisciplinary approach. However, within available treatments for chronic muscle pain, there is a paucity of studies about the effectiveness of TP needling in masticatory muscles, thus giving rise to our objective of studying the DDN of myofascial TPs in the LPM. The mechanism of inactivation of a TP by needling is unknown, although we consider that the presence of a LTR during DDN is important given its proven relationship with the desired therapeutic effect (3,4,13,15-21).

Since stretching techniques and massage are difficult and complicated by the limited spatial access to the LPM, needling of TPs may be necessary (3,4,9). The critical importance of this muscle as origin of temporomandibular myofascial pain makes acquisition of the skills necessary for performing TP needling a worthwhile exercise. The external approach (extra oral DDN) permits needling of the central TPs in the muscle bellies of the two divisions of the muscle and of the insertional TPs of the posterior myotendinous junctions of both divisions (3,4). Determining the correct location of the muscle mass is essential for the technique we use, so interfering factors represented by the nearby bony structures (zygomatic arch, coronoid process, condyle and sigmoid notch of the mandible) must be circumvented. There seems to be no need to carry out the electromyographic control (22), given the TPs can be detected by following the diagnostic criteria already described and by obtaining a LTR during needling.

The objective of the present study was to evaluate the efficacy of three applications of such needling of the LPM on a once per week basis for three weeks. In this way, a comparison was made of pain intensity at rest and upon mastication, as well as measurements of ranges of maximum mouth opening, laterality, and protrusion movements. The results showed statistically significant differences in favor of DDN compared with respect to an oral methocarbamol/paracetamol combination therapy. Neither treatment was superior to the other concerning the limitation of mandibular opening. In the DDN-treated patients, parameters were significantly improved in both follow-up visits (days 28 and 70) compared to day 0. This was not the case for the control group, where only pain on day 28 was statistically significantly improved. When we evaluated the functionality of the TMJ, with the 100-point test, the DDN treatment approach was clearly superior. Similarly, the overall assessment by patients and authors of the effectiveness of the treatment, and the evaluation of treatment tolerance by patients, was better with the three applications of DDN than with methocarbamol/paracetamol combination treatment. Pain reduction in the DDN-treated group was greater the higher was pain intensity at baseline (day 0). The global evaluation of efficacy as assessed both by patients and authors was better for the DDN test group. No adverse reactions were detected with DDN, whereas up to 41% of the patients receiving the combination drug treatment described unpleasant side effects (drowsiness).

This study has some limitations. First, treatment appraisal was limited only to the effects seen in the short- and mid-term (eight weeks following completion of DDN of TPs in the LPM). A study with a larger size sample and a longer follow-up period is required to determine the long-term benefits of DDN of TPs in this muscle. A second limitation is that the study was not a real doubled-blind and included an active treatment comparison group with methocarbamol/paracetamol, as a control group. A third limitation is that the control group had a significant number of withdrawal study subjects (8 patients), with the main reason for dropping out being due to personal difficulties associated with patients keeping their scheduled appointments.

To conclude, DDN of TPs in the LPM showed better efficacy and safety in reducing pain and improving maximum mouth opening, laterality, and protrusion movements in patients with chronic myofascial pain located on that muscle, compared with an oral methocarbamol/paracetamol combination therapy. The improvement persisted for 8 weeks following completion of the treatment, and was proportional to the intensity of the pain at the beginning of the study. Our study also suggested that patients with a poorer functional status prior to treatment obtained the best final outcomes. No serious adverse events were observed with respect to the dry needling technique.
